# Coal Gangue Recycling in Construction Materials: Strategies for Environmental Risk Mitigation via Heavy Metal Immobilization and Resource Utilization

**DOI:** 10.3390/ma19050949

**Published:** 2026-02-28

**Authors:** Yang Xue, Xiaoming Liu, Xuchao Wang, Wei Zhang

**Affiliations:** 1 State Key Laboratory of Advanced Metallurgy, University of Science and Technology Beijing, Beijing 100083, China; 2 School of Metallurgical and Ecological Engineering, University of Science and Technology Beijing, Beijing 100083, China; 3 Department of Civil Engineering, Hebei Agricultural University, Baoding 071001, China

**Keywords:** coal gangue, heavy metal, recycling, utilization, sustainable

## Abstract

Coal gangue represents the predominant solid waste in the coal industry and poses significant risks to both the ecological environment and human health. It has been demonstrated that recycling it in building materials effectively reduces stockpiling, mitigates environmental harm, and minimizes heavy metal leaching. However, a comprehensive review systematically focusing on the recycling of coal gangue and the behavior of its associated heavy metals in building materials is still lacking. This work introduces the physicochemical properties and environmental hazards of coal gangue, including spontaneous combustion, land occupation, and pollution risks. It also summarizes the leaching patterns, speciation, and immobilization mechanisms of heavy metals such as Cr, Cu, and Pb in gangue-based building materials, and reviews adsorption behaviors, solidification pathways, and microstructural interactions at the molecular scale. Despite ongoing efforts, over five billion tons of coal gangue remain accumulated in China, with secondary pollution from heavy metals continuing to pose serious concerns. To address these challenges, recommendations are proposed for establishing standardized leaching evaluation methods, and a novel approach for transitioning from heavy metal solidification to active utilization is introduced. This review aims to provide strategic direction for the green and sustainable recycling of coal gangue.

## 1. Introduction

**Figure 1:**
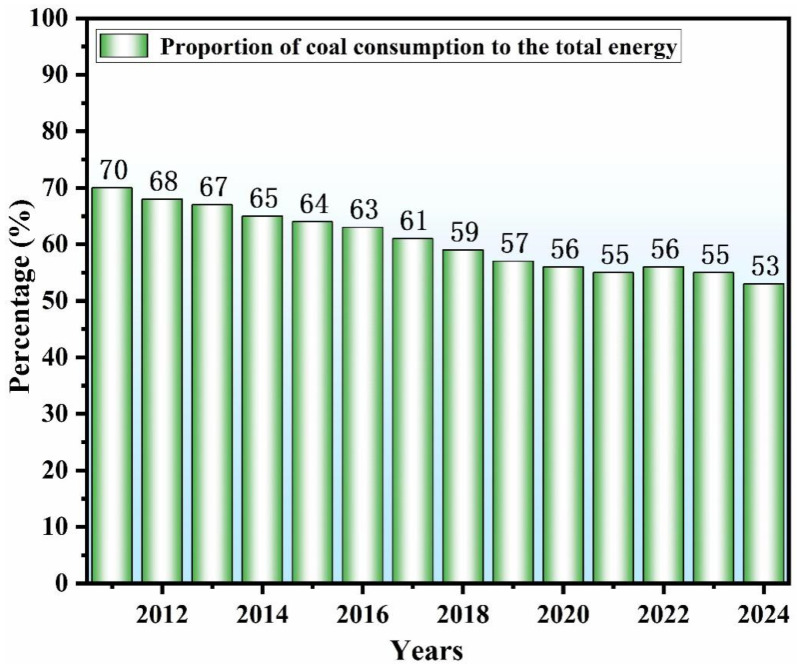
Proportion of coal consumption to the total energy in 2011–2024 [12].

Coal has long been a major global energy source, having contributed substantially to worldwide industrial progress. In 2024, global consumption reached approximately 5.32 billion tons, accounting for over 19% of total global primary energy consumption [1]. As shown in Figure 1, while the proportion of coal in China’s energy structure has declined since 2011, it still represented more than half of the national total in 2024 [2]. However, the sustainability of this energy pathway is increasingly challenged by the environmental impacts of its extraction and use [3]. The mining and processing of coal generate large amounts of gangue, a hazardous solid waste accounting for an estimated 10–15% of raw coal output [4,5]. In China, the historical accumulation of gangue is reported to exceed 5 billion tons, with annual additions continuing at around 350 million tons [6]. This extensive stockpiling occupies considerable land and triggers multiple environmental issues. For instance, heat accumulation within stockpiles can lead to spontaneous combustion, releasing large quantities of hazardous gases—including sulfur dioxide, nitrogen oxides, and carbon monoxide—into the atmosphere [7,8]. Furthermore, improper stacking increases the risk of geological hazards such as debris flows and landslides, threatening both the environment and public safety [9,10]. Long-term stockpiling also allows heavy metals to leach out and infiltrate both surface and groundwater systems, posing persistent risks to ecological balance and human health [11]. Therefore, improving the utilization rate of this industrial byproduct and mitigating its associated hazards is imperative.

Over the past two decades, global efforts have been made to utilize coal gangue across various sectors, aiming to mitigate its accumulation and associated environmental impacts. For instance, valuable components such as aluminum, lithium, iron, and gallium have been extracted from it [13,14], and it has been employed in preparing catalysts for chemical and biological processes [15,16,17]. Due to its content of silicon, phosphorus, and potassium, coal gangue has also been used in developing plant fertilizers [18,19].

Several reviews have summarized aspects of coal gangue utilization. For example, Li [6] discussed its integrated use and environmental hazards, while Guo et al. [20] analyzed the utilization potential of this energy-associated waste. Despite these diverse applications, the actual consumption rate of coal gangue remains low. A persistent concern is the serious pollution of the ecological environment by harmful heavy metals released from gangue [21].

Research indicates that incorporating coal gangue into building materials represents one of the most feasible routes for its large-volume consumption [22,23]. This approach not only helps reduce CO_2_ emissions from the cement industry but also mitigates the environmental hazards posed by gangue stockpiling [24,25]. Moreover, the high silicon and aluminum content in coal gangue can be readily activated by alkali to form silica–alumina gel phases, which significantly enhance the performance of the resulting building materials [26,27]. Therefore, investigating the high-dosage application of coal gangue in building materials and exploring innovative methods for its use are crucial for advancing green and low-carbon development in the construction sector [28].

Although significant progress has been made in utilizing coal gangue for building materials, a systematic review focusing specifically on coal gangue-based building materials is still lacking. Importantly, a comprehensive summary of the environmental hazards of coal gangue and the immobilization mechanisms for its heavy metals is needed.

In this work, we review and analyze a substantial body of literature pertaining to coal gangue. We begin by outlining current trends and potential utilization pathways. The physicochemical properties and environmental hazards of coal gangue are introduced. We then critically review the leaching behavior and immobilization (solidification) of heavy metals in coal gangue-based building materials, summarizing the various underlying mechanisms. Based on this analysis, we identify the key challenges in the current application of coal gangue and propose scientific recommendations. This review aims to serve as a reference for advancing the application of coal gangue and controlling its heavy metals, thereby stimulating innovative thinking for the efficient resource utilization of this industrial solid waste.

## 2. Physicochemical Properties of Coal Gangue

### 2.1. Physical Properties

Coal gangue has a density ranging from 1.5 to 2.7 g/cm^3^, which is approximately 80–90% that of raw coal [29]. It exhibits high porosity (45–60%), with its water content increasing proportionally with porosity [30]. The presence of sulfur and iron compounds renders it prone to oxidation, often leading to cracking and pulverization [31]. Its compressive strength, ranging from 10 to 30 MPa [32], indicates high hardness and consequent handling difficulty. The original particle size of coal gangue varies widely from 5 to 150 mm, allowing for the selection of appropriate pretreatment methods based on the specific particle size distribution [33].

### 2.2. Chemical Composition

The chemical composition of coal gangue, as compiled from various sources in the literature, is summarized in Table 1. Major coal gangue deposits in China are concentrated in provinces such as Shanxi, Hebei, Liaoning, and Shandong. Although the specific mass percentages of constituents vary by region, the chemical composition is consistently dominated by similar oxides, primarily SiO_2_, Al_2_O_3_, CaO, Fe_2_O_3_, Na_2_O, MgO, TiO_2_, K_2_O, SO_3_ [34,35,36,37,38,39,40,41,42,43,44,45,46,47,48]. Notably, the combined content of SiO_2_ and Al_2_O_3_ ranges from 56.49% to 92.13% by mass. This high proportion confirms that coal gangue is a silica–alumina-rich material, endowing it with potential pozzolanic activity for use in building materials.

As shown in Table 1, the loss on ignition (LOI) of coal gangue exceeds 6% in samples from Datong, Pingshuo, Wuhan, Shijiazhuang, and Xuzhou. Notably, in Wuhan, Datong, and Pingshuo, the LOI values are above 27%. This is attributed to the presence of residual carbon and volatile matter in the coal gangue.

### 2.3. Mineral Phases

The primary sources of the useful SiO_2_ and Al_2_O_3_ components in coal gangue are kaolinite. Structurally, this triclinic silicate mineral is characterized by the stacking of Al–O octahedral and Si–O tetrahedral sheets along the axis. The connectivity is maintained by Al^3+^ ions coordinated with four intralayer hydroxyl groups and two bridging oxygen atoms that link the octahedral and tetrahedral layers together [49]. Simulated crystal structures reveal a network of stable hydrogen bonds both within and between kaolinite crystals. This stable crystalline arrangement severely limits the penetration of water and other molecules into the lattice, accounting for kaolinite’s relatively low hydration reactivity. Consequently, enhancing the reactivity of its silica–alumina components require disrupting this layered crystal structure [50].

### 2.4. Heavy Metal Content

Heavy metals are recognized as hazardous constituents in coal gangue, posing significant risks to ecosystems and human health. Their concentrations vary considerably across different geographical regions. Analytical data from mining areas in Chongqing, Yunnan, Anhui, and other locations indicate the following content ranges (in mg/kg): arsenic (As), 0.08–20.6; lead (Pb), 0.49–33.45; chromium (Cr), 11.42–399.20; cadmium (Cd), 0.08–0.30; nickel (Ni), 24.31–69.33; copper (Cu), 24.07–41.80; manganese (Mn), 157.01–776.90; mercury (Hg), 0.01–0.04; and zinc (Zn), 76.27–123.21 [4,47,51,52,53,54,55]. Among these, As, Pb, Cr, Ni, Cu, Mn, and Zn generally occur at higher concentrations, while Cd and Hg are typically present at lower levels [52].

## 3. Environment Hazards and Risks of Coal Gangue

The primary hazards arising from coal gangue accumulation include spontaneous combustion, ecological impacts, water pollution, and geological disasters. In particular, heavy metals released from coal gangue pose significant threats to natural ecosystems and human health [56]. These hazards are further manifested through atmospheric and groundwater pollution, which can lead to adverse effects on wildlife and human diseases. The specific manifestations of coal gangue-related harm are summarized as follows:

### 3.1. Spontaneous Combustion and Atmospheric Pollution

According to statistics, there are over 2000 coal gangue hills in China’s coal mining areas, approximately one-third of which undergo spontaneous combustion [57]. The process involves the ignition of residual coal and other combustibles within the gangue (the ignition point of coal typically ranges around 360 °C) at temperatures between 800 and 1000 °C. This combustion releases harmful gases such as CO, CO_2_, SO_2_, H_2_S, and NO_x_ [58]. Consequently, the air quality around gangue hills and the health of nearby residents are adversely affected by these emissions.

### 3.2. Land Occupation and Ecological Degradation

Currently, coal gangue stockpiles in China cover an estimated area of approximately 1.5 × 10^8^ m^2^, with an annual growth of about 4 × 10^6^ m^2^. The accumulation of coal gangue occupies substantial land space and damages the original topography and vegetation [59]. Additionally, dust generated from gangue piles contributes to air pollution, further degrading the natural environment of mining areas.

### 3.3. Water Pollution via Leaching

When coal gangue remains submerged for extended periods, harmful components can leach out, leading to the contamination of surrounding water and soil. The acidic leachate, upon entering water bodies, inhibits microbial growth and impairs the water’s self-purification capacity [60]. Furthermore, coal gangue contains toxic heavy metals such as As, Pb, Cr, and Cd, which can seriously pollute the aquatic environment and affect aquaculture [61].

### 3.4. Risks of Geological Disasters

Coal gangue hills are typically characterized by steep slopes, loose structures, and inadequate stabilization measures. The natural angle of repose for coal gangue—the maximum slope angle at which the material remains stable—ranges from 38° to 40°. Disasters such as collapses, landslides, and debris flows can be triggered by excessive pile height, over-steepened slopes, excavation activities, blasting, and heavy rainfall erosion [62,63]. Consequently, geological disasters frequently occur in areas with coal gangue accumulations in China.

## 4. Leaching and Solidification/Stabilization of Heavy Metals

### 4.1. Leaching Behavior and Assessment of Heavy Metals

#### 4.1.1. Leaching from Artificial Aggregates

Experimental results indicate that the leaching concentrations of Cr, Cu, and Pb from all coal gangue-based artificial aggregates ranged from 0.025–0.068 mg/L, 0.038–0.081 mg/L, and 0.021–0.034 mg/L, respectively. These findings confirm that the leaching levels of copper, chromium, and lead comply with the safety limits specified in the Chinese National Standard GB 5085.3-2007 [64]. Furthermore, the immobilization capacity of these aggregates for heavy metals is significantly influenced by the sintering temperature and the dosage of feldspar powder (FP) [37].

#### 4.1.2. Immobilization Capacity of Binders with Coal Gangue

Analysis of the consolidation efficiency reveals that the immobilization rates of Cr, Zn, Cu, and Pb within the binder matrix all exceed 85%. This high efficiency demonstrates that the incorporation of coal gangue enhances the binder’s capacity to sequester harmful heavy metal ions. This performance is attributed to the development of a dense pore structure in these high-performance materials, which restricts the diffusion of leaching fluids. Additionally, heavy metals are chemically stabilized through incorporation into hydration products, such as C-(A)-S-H and N-A-S-H gels, within the matrix [65,66,67]. The synergy between these physical encapsulation and chemical bonding mechanisms strengthens the overall consolidation capacity of gangue-based materials [68].

#### 4.1.3. Conceptual Modeling for Heavy Metal Release

To elucidate the release process, a leaching mechanism model was developed considering the complex interactions between coal gangue and its environment [55,69]. This theoretical framework evaluates the individual effects and synergistic interactions of particle size, leachate pH, iron ion concentration, and microbial activity [70,71,72,73,74]. In this process, the leachate acts as the primary reaction medium. Particle size determines the effective contact area for solute-solvent interactions and catalytic processes involving Fe^3+^ and ferrous oxides. The oxidative efficacy of these iron species is highly sensitive to the pH of the leaching solution. The positive synergy among these four parameters suggests that the environmental hazard posed by heavy metals in coal gangue dumps can increase exponentially over time if left unmanaged.

#### 4.1.4. Ecological Risk Assessment

The environmental hazards of heavy metals in soils impacted by coal gangue were evaluated using the Potential Ecological Risk Index (PERI). The calculated average PERI values for Cu (0.85), Zn (4.66), Cr (5.63), and Ni (6.48) are all well below the threshold of 40, indicating that the overall potential leaching risk from the coal gangue-amended soil is low.

However, the mobility of these metals warrants attention. Assessment via the Risk Assessment Code (RAC), based on acid-extractable fractions, shows that the RAC values for Mn, Fe, Cr, Zn, Ni, and Cu in the 0–40 cm soil layer all exceed 1%. This indicates a non-negligible potential for migration and release [75].

The overall leaching risk at disposal sites is governed by metal speciation, physicochemical properties, and soil characteristics. This chronic, cumulative process involves: (1) initial input via rainfall infiltration and atmospheric deposition; (2) transformation into non-residual, bioavailable forms with high mobility; and (3) long-term toxicity accumulation. To mitigate these risks, practical measures such as installing geotextile liners in sanitary landfills and constructing drainage channels to intercept leachate are essential [75].

Leaching toxicity testing of heavy metals in coal gangue is essential for assessing its potential risks to the environment and human health. These experiments identify and quantify the leachable fractions of heavy metals, thereby elucidating their potential toxic effects. Consequently, leaching studies play a crucial role in guiding the management and utilization of coal gangue. This section summarizes current research on the leaching behavior of heavy metals from coal gangue and the associated predictive models.

### 4.2. Existential State of Heavy Metals in Coal Gangue-Based Building Materials

#### 4.2.1. Coal Gangue-Based Geopolymer Materials

Heavy metals within coal gangue-based geopolymers are consolidated through a combination of physical encapsulation and chemical bonding. Physically, heavy metal ions are effectively trapped within the complex three-dimensional network of the geopolymer gel. Simultaneously, the gel coats the surfaces of unreacted fly ash (FA) and bottom ash (BA) particles, creating a barrier that inhibits leaching [76]. From a chemical perspective, phase and chemical bond analyses confirm that various heavy metal salts precipitate directly within the geopolymer matrix. Furthermore, the “ion pair effect” plays a significant role: larger heavy metal ions tend to form weaker ion pairs within the geopolymer structure, which facilitates their integration with silicate species [77]. For example, Pb^2+^ ions can readily replace Na^+^ or K^+^ ions within the geopolymer framework, leading to stable chemical incorporation [78].

#### 4.2.2. Coal Gangue-Based Adsorbed Functional Building Materials

Functional adsorbent building materials produced from coal gangue are effective for removing heavy metals from wastewater [79]. However, it is critical to ensure these materials do not become secondary sources of pollution through their own leaching [80]. To manage this risk, an assessment system based on the Chinese standard GB 30760-2014 [81] has been established. Based on integrated test data, the following leaching thresholds are proposed for safety: As ≤ 6.0 mg/kg, Pb ≤ 0.3 mg/kg, and Cr ≤ 2.0 mg/kg.

#### 4.2.3. Coal Gangue-Based Architectural Ceramics Materials

The chemical states of lead (Pb) and zinc (Zn) introduced into architectural ceramics (samples CP3.0 and CZ3.0) were analyzed using X-ray photoelectron spectroscopy (XPS). The recorded binding energy peaks at 136.82 eV (Pb 4f_7/2_) and 141.63 eV (Pb 4f_5/2_) confirm that Pb exists in the divalent state (Pb^2+^) within the ceramic matrix [82]. Similarly, peaks at 1021.30 eV (Zn 2p_3/2_) and 1044.92 eV (Zn 2p_1/2_) verify the presence of Zn^2+^ [83]. Since the valence states remain unchanged compared to their nitrate precursors, it is evident that these metals are successfully incorporated into the structure without redox transformation.

Scanning electron microscopy with energy-dispersive spectroscopy (SEM-EDS) further reveals that both elements are distributed homogeneously throughout the ceramic matrix. While some isolated macropores may show an absence of these metals, the general uniformity suggests that effective encapsulation occurs during the high-temperature sintering process. Consequently, physical encapsulation within the glassy and crystalline phases is the primary mechanism for heavy metal immobilization in these ceramics.

#### 4.2.4. Coal Gangue-Based Soil Materials

Spatial distribution analysis using inverse distance weighting interpolation reveals a clear correlation between heavy metal concentrations and proximity to coal gangue disposal sites. For elements such as Cd, Cr, Ni, Cu, Zn, and As, significantly higher concentrations are observed in areas adjacent to gangue stockpiles [84,85,86]. This pattern indicates that leaching and weathering of the gangue are the primary drivers of soil contamination, as rainfall runoff transports metals into the surrounding environment.

Additionally, elevated metal levels at certain sampling points suggest that atmospheric deposition also contributes to surface soil accumulation. Therefore, leaching/runoff and atmospheric deposition are identified as the two dominant pathways for heavy metal enrichment. While other sources like fertilizers and irrigation water may contribute to the baseline [87,88,89], accurate source apportionment remains essential for targeted soil remediation and pollution control [90].

#### 4.2.5. Coal Gangue-Based Artificial Aggregates

The micro-morphology and mineralogical evolution of coal gangue-based artificial aggregates explain their high stability. During sintering, a substantial glass phase forms that effectively encapsulates heavy metals. More importantly, long-term stabilization is achieved as heavy metals transition from mobile ionic states into stable crystalline forms, significantly reducing their leaching potential [91].

Mineral composition analysis reveals that in the C1G4F5 mixture (10% clay, 40% coal gangue, 50% feldspar powder), heavy metals are converted into stable crystalline phases upon sintering at 1170 °C, including plumbum aluminosilicate (5PbO·Al_2_O_3_·10SiO_2_), Luddenite (CuPbSi_5_O_14_·14H_2_O), and Natalyite (NaCrSi_2_O_6_). Existing studies indicate that Cr^3+^ in Natalyite tends to form more stable compounds compared to Cr^6+^ [92].

In summary, this subsection has described the immobilization states of heavy metals in coal gangue-based building materials. The key consolidation mechanisms include: physical encapsulation, physical adsorption, chemical ion binding effects, the formation of new heavy-metal-containing compounds, and the crystallization of heavy metal ions. Accurately quantifying the sources and speciation of heavy metals is fundamental to further improving their consolidation efficiency.

### 4.3. Solidification Mechanisms of Heavy Metal from Coal Gangue

#### 4.3.1. Adsorption Mechanism of Heavy Metals

**Figure 2:**
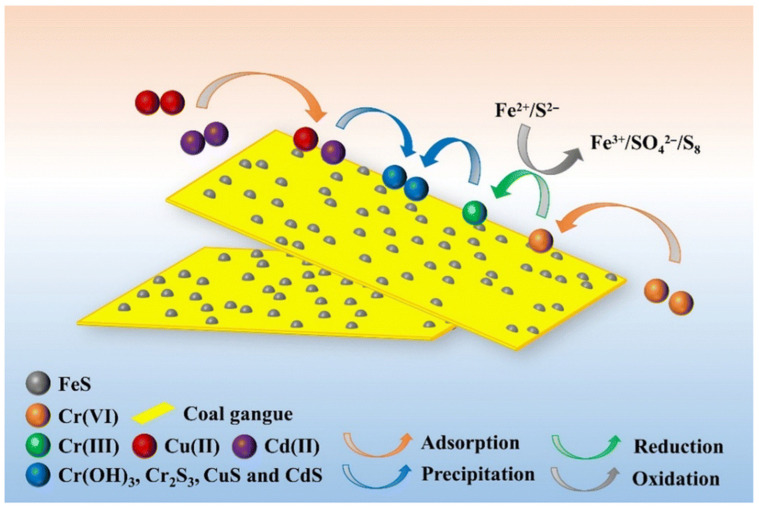
Adsorption mechanism diagram of Cr (VI), Cr (III), Cu (II) and Cd (II) by nFeS-CG [90].

Heavy metals (e.g., Cu^2+^) and organic contaminants (e.g., Rhodamine B, Rh-B) in coal gangue can be effectively sequestered by a composite structure of zeolite and activated carbon. In this system, Cu^2+^ ions are primarily accommodated within the precise micropores of the zeolite framework. Conversely, because the average pore size of the hierarchical structure in activated carbon is significantly larger than the ionic diameter of Cu^2+^, these ions are more prone to escape from the larger carbon pores. As illustrated in Figure 2, the removal mechanism of Cr (VI), Cu (II), and Cd (II) by the coal gangue-derived nFeS-CG involves a synergistic sequence of electrostatic adsorption, redox reduction, and chemical precipitation. Under acidic conditions, Cr (VI) (as HCrO_4_^−^) is rapidly adsorbed via electrostatic attraction onto the protonated surface and reduced to Cr (III) by ionized Fe^2+^ and S^2−^. As H^+^ is consumed and the pH rises, the process shifts toward the adsorption of Cu (II) and Cd (II), culminating in the formation of insoluble sulfide and hydroxide precipitates—such as Cr_2_S_3_, CuS, CdS, and Cr(OH)_3_—on the material surface [90].

**Figure 3:**
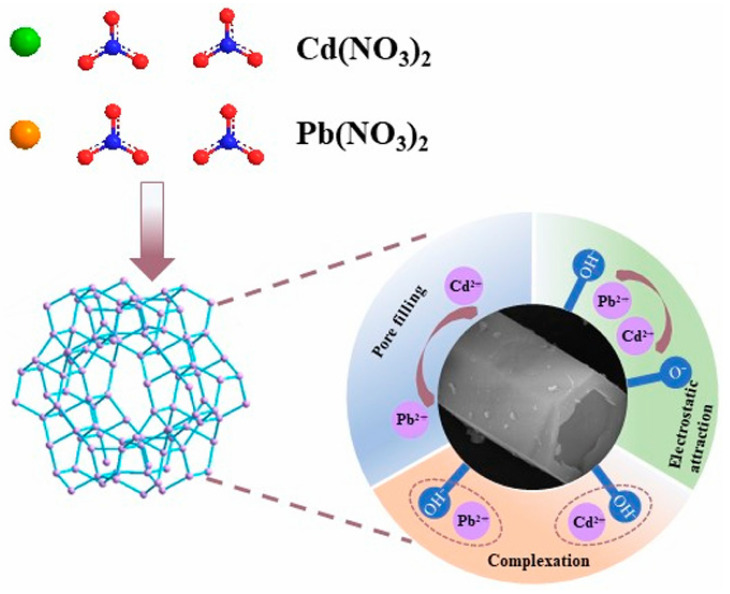
Schematic of the adsorption mechanism of heavy metal ions by coal gangue-based SAPO-5 molecular sieve [95].

The composite material exhibits significantly higher adsorption efficiency for organic pollutants like Rh-B compared to pure zeolite. Organic molecules with diameters exceeding the zeolite pore capacity are adsorbed onto its external surface, while larger organic macromolecules are effectively captured by the multilayer pore structure of the activated carbon. Furthermore, the introduction of activated carbon induces structural defects and dislocations in the zeolite framework, increasing the proportion of mesopores and macropores. This expanded pore volume provides additional storage space, allowing the zeolite/activated carbon composite to achieve an overall adsorption efficiency of 94.2% for heavy metals and organic pollutants [93,94]. As illustrated in Figure 3, the adsorption mechanism of heavy metal ions by the coal gangue-derived SAPO-5 zeolite is primarily driven by the synergistic effect of pore filling—enabled by its 2.03 nm mesoporous structure—and electrostatic attraction, which is significantly modulated by the solution pH [95].

#### 4.3.2. Gray-Scale Evaluation for the Consolidation Mechanism of Heavy Metals

The correlation between ash composition and heavy metal immobilization can be quantified using grey relational analysis. Analysis of the chemical components reveals that CaO, SiO_2_, Al_2_O_3_, Fe_2_O_3_, and P_2_O_5_ exhibit high relational grades with the efficient consolidation of heavy metals. Given that both coal gangue and sludge are rich in SiO_2_ and Al_2_O_3_, their complementary chemical profiles synergistically facilitate the immobilization of Pb, Cd, As, and Cr.

Combined with mineralogical characteristics, physical interactions between minerals such as gabbro (Ca_2.86_Mg_0.14_PO_4_)_2_) and diopside (CaMgSi_2_O_8_) can hinder heavy metal volatilization by reducing combustion rates or altering chemical bonding pathways. For instance, the transformation of aluminosilicate crystal structures can induce charge transfer effects, further promoting the long-term stabilization of heavy metals [96].

#### 4.3.3. Microstructure About Heavy Metals Consolidation

The immobilization behavior of heavy metals is deeply rooted in the physicochemical properties of coal gangue-based materials. SEM-EDS analysis confirms the adsorption of Cd, Cu, and Pb onto these materials. Surface examination shows significant precipitation on samples treated with 2% H_3_PO_4_-modified carbon hydroxide (BPH), whereas no such precipitation is detected on pristine Na-X zeolite (ZL) used as a control. When a combined treatment (1% BPH + 1% ZL) is applied, precipitation becomes evident on both surfaces, indicating that mineral precipitation—likely induced during leaching or washing—is a key immobilization mechanism contributed by the BPH component.

Furthermore, EDS data revealed that the adsorption affinity for the three heavy metals followed the order: Pb > Cu > Cd. According to previous studies, this competitive adsorption is governed by ionic properties such as hydrated radius, electronegativity, and hydrolysis constant [97]. For divalent cations, the order of hydrolysis constants and electronegativity is Pb^2+^ > Cu^2+^ > Cd^2+^. Generally, heavy metals with higher hydrolysis constants and electronegativity exhibit stronger adsorption affinity. Additionally, the smaller hydrated radius of Pb^2+^ (0.401 nm), compared to Cd^2+^ (0.426 nm) and Cu^2+^ (0.419 nm), further facilitates its adsorption onto the material surface [98].

#### 4.3.4. Coupling Mechanism of Heavy Metal Consolidation

The immobilization states of As, Pb, and Cr in coal gangue involve complex binding states with aluminosilicates and iron oxides, as well as adsorption on the surfaces of clay and other mineral particles. Free heavy metals in solution can be precipitated or adsorbed by iron (hydr)oxide colloids following changes in acidity. Additionally, trace heavy metals may form secondary minerals through ion-exchange processes. Despite these immobilization pathways, the inherent mobility of heavy metals remains significant, leading to potential contamination of water systems, aquatic organisms, and soil [93].

#### 4.3.5. Molecular Scale Analysis of Heavy Metal Consolidation

Density functional theory (DFT) calculations reveal that the binding of heavy metals to kaolinite surfaces is primarily mediated by its hydroxyl functional groups [99]. Heavy metals exhibit a strong tendency to bind to hydroxyl groups on the (001) surface, whereas the affinity for hydroxyls on the (001) surface is weaker. On the α-quartz (001) surface, heavy metals are mainly adsorbed via electrostatic interactions with oxygen atoms in the silica framework. In contrast, adsorption on the anatase (001) surface involves a combination of van der Waals forces and electrostatic attraction.

Within silicate minerals, heavy metals can be incorporated into the crystal lattice through precipitation and complexation. Notably, higher binding energies between heavy metals and mineral surfaces correspond to more stable immobilization, resulting in lower mobility and release potential. These computational insights align with experimental extraction data for six heavy metals from Hongxiang coal gangue. Arsenic (As) exhibited the strongest binding energies (51.95, 46.36, 43.22, and 35.90 eV) and the lowest migratory fraction (2%). In comparison, lead (Pb) showed higher migration potential (0.49 mg/kg) and lower binding energies (20.24, 16.49, 15.58, and 14.71 eV) [53].

In summary, multiple mechanisms contribute to the immobilization of heavy metals from coal gangue in building materials. These include (1) physical adsorption within pores of varying sizes and through microstructural charge equilibria [100]; (2) chemical incorporation into new mineral phases via component consolidation and ion exchange; (3) surface adsorption mediated by van der Waals forces and electrostatic attraction; (4) lattice incorporation through precipitation and complexation within silicate minerals.

## 5. Conclusions and Suggestions

The discharge of coal gangue has increased sharply due to the rapid growth in global energy demand. Its accumulation occupies vast land resources, pollutes the natural ecological environment, and poses risks to human health. Currently, the harmless treatment and resource utilization of coal gangue face considerable challenges. Previous research has demonstrated that utilizing coal gangue to prepare building materials and immobilize its heavy metals is an important strategy for reducing stockpiling and mitigating environmental pollution. This review introduces the physicochemical properties and environmental hazards of coal gangue. It summarizes the leaching patterns and speciation of heavy metals in various coal gangue-based products, including geopolymers, wastewater treatment materials, architectural ceramics, soil amendments, and artificial aggregates. The immobilization characteristics and underlying mechanisms are also reviewed. However, the market competitiveness of coal gangue-based building materials remains limited due to their inherent properties, processing requirements, and perceived technical barriers. Despite some progress in recycling, the stockpile of coal gangue remains enormous, and secondary pollution from its heavy metals continues to be a serious concern.

Therefore, the large-scale utilization of coal gangue and the effective immobilization of its heavy metals require further in-depth research prior to widespread practical application. While laboratory-scale research has successfully validated immobilization mechanisms such as physical encapsulation and chemical bonding, a substantial gap remains before these technologies can be directly implemented at an industrial scale. Laboratory experiments typically utilize homogenized samples under precise control, whereas industrial production must address the high heterogeneity of large-volume coal gangue and ensure the energy efficiency of large-scale sintering or activation equipment to maintain economic viability. Future realization depends on transitioning from static laboratory leaching tests to dynamic monitoring systems that account for real-world factors like aeolian erosion, heavy rains, and floods, while shifting research focus toward “active utilization” where heavy metals are integrated into new mineral phases to enhance the functional properties of building materials. Bridging this gap through pilot-scale trials will be essential to validate the consistency and long-term durability of these recycled materials in fluctuating field environments before widespread application can be achieved. In view of the aforementioned challenges, the following recommendations are proposed:

(1) Although research on traditional immobilization methods has progressed, over 5 billion tons of coal gangue remain accumulated. This review proposes innovative approaches, such as utilizing heavy metals within gangue to form new mineral phases that enhance the properties of building materials.
(2) Discharge and utilization standards for coal gangue should be established based on its specific characteristics across different industries, enterprises, and regions. Policies promoting technological innovation for its efficient use should be implemented to guide its correct and safe application.
(3) While numerous application technologies exist, safety and environmental protection are paramount. Traditional leaching risk assessments are often inadequate; therefore, intelligent systems for the quantitative tracking of heavy metals need to be developed to provide critical data for scientific management.
(4) New avenues for large-scale, cross-sectoral recycling should be explored. This requires a comprehensive consideration of the physicochemical properties of coal gangue to achieve energy savings or waste heat recovery across various processes, moving beyond single-industry utilization.

## Figures and Tables

**Table 1:** Chemical composition of raw coal gangue sourced from regions and cities (wt.%).

Sources	SiO_2_	Al_2_O_3_	CaO	Fe_2_O_3_	Na_2_O	MgO	TiO_2_	K_2_O	SO_3_	LOI	Ref.
Shanxi	53.50	24.30	0.5	9.80	0.4	0.50	1.1	1.90	7.90	0.10	[34]
Shanxi	53.32	24.32	0.46	6.76	0.39	0.45	1.05	1.69	7.78	3.78	[35]
Yulin, Shanxi	57.98	21.88	4.30	6.01	1.22	1.22	1.57	3.11	1.91	0.81	[36]
Datong, Shanxi	47.49	17.36	1.68	1.76	1.04	0.75	0.75	1.69	-	27.35	[37]
Pingshuo, Shanxi	34.74	29.57	0.71	3.85	0.05	0.13	0.94	0.20	-	29.44	[38]
Xinzhou, Shanxi	48.80	22.49	3.57	11.12	-	1.72	1.12	1.96	7.42	1.80	[39]
Shijiazhuang, Hebei	51.76	40.37	2.43	0.57	0.24	0.24	0.35	0.21	-	0.81	[40]
Shijiazhuang, Hebei	42.01	43.15	0.65	2.98	-	0.54	1.03	1.21	-	8.43	[41]
Fuxin, Liaoning	58.42	17.31	3.48	7.50	1.60	8.42	0.95	3.92	0.65	-	[42]
Fuxin, Liaoning	58.81	20.66	3.69	8.15	0.68	2.31	1.15	3.31	0.30	0.93	[43]
Huafang, Shandong	52.10	28.30	8.30	7.20	-	4.10	-	-	-	-	[44]
Wuhan, Hubei	29.11	27.38	0.34	2.43	0.02	0.08	0.90	0.08	4.99	30.01	[45]
Xuzhou, Jiangsu	61.69	19.11	2.35	4.16	2.28	0.64	-	3.04	-	6.73	[46]
Chongqing	51.56	25.83	3.35	6.32	-	0.64	3.14	1.31	6.49	1.36	[47]
Jerada	52.4	21.9	0.81	4.55	1.53	1.26	-	2.24	3.54	0.01	[48]

## Data Availability

No new data were created or analyzed in this study.
